# Well-known surface and extracellular antigens of pathogenic microorganisms among the immunodominant proteins of the infectious microalgae *Prototheca zopfii*

**DOI:** 10.3389/fcimb.2015.00067

**Published:** 2015-09-29

**Authors:** Alexandra Irrgang, Jayaseelan Murugaiyan, Christoph Weise, Walid Azab, Uwe Roesler

**Affiliations:** ^1^Institute of Animal Hygiene and Environmental Health, Centre for Infection Medicine, Freie Universität BerlinBerlin, Germany; ^2^Institute for Chemistry and Biochemistry, Freie Universität BerlinBerlin, Germany; ^3^Institute of Virology, Centre for Infection Medicine, Freie Universität BerlinBerlin, Germany

**Keywords:** *Prototheca*, canine protothecosis, immunodominant proteins, western blotting, proteomics, MALDI TOF MS

## Abstract

Microalgae of the genus *Prototheca* (*P*.) are associated with rare but severe infections (protothecosis) and represent a potential zoonotic risk. Genotype (GT) 2 of *P. zopfii* has been established as pathogenic agent for humans, dogs, and cattle, whereas GT1 is considered to be non-pathogenic. Since pathogenesis is poorly understood, the aim of this study was to determine immunogenic proteins and potential virulence factors of *P. zopfii* GT2. Therefore, 2D western blot analyses with sera and isolates of two dogs naturally infected with *P. zopfii* GT2 have been performed. Cross-reactivity was determined by including the type strains of *P. zopfii* GT2, *P. zopfii* GT1, and *P. blaschkeae*, a close relative of *P. zopfii*, which is known to cause subclinical forms of bovine mastitis. The sera showed a high strain-, genotype-, and species-cross-reactivity. A total of 198 immunogenic proteins have been analyzed via MALDI—TOF MS. The majority of the 86 identified proteins are intracellularly located (e.g., malate dehydrogenase, oxidoreductase, 3-dehydroquinate synthase) but some antigens and potential virulence factors, known from other pathogens, have been found (e.g., phosphomannomutase, triosephosphate isomerase). One genotype-specific antigen could be identified as heat shock protein 70 (Hsp70), a well-known antigen of eukaryotic pathogens with immunological importance when located extracellularly. Both sera were reactive to glyceraldehyde-3-phosphate-dehydrogenase of all investigated strains. This house-keeping enzyme is found to be located on the surface of several pathogens as virulence factor. Flow-cytometric analysis revealed its presence on the surface of *P. blaschkeae*.

## Introduction

*Prototheca spp*. are inconspicuous microorganisms that have remained poorly described despite their worldwide distribution. Nevertheless, these unicellular algae attracted our attention because of their ability to infect vertebrates with severe manifestations. Since its discovery in 1894 by Krüger, the taxonomic classification of *Prototheca* has been discussed controversially (Krüger, 1894). On the one hand, because of their yeast-like appearance when cultured on Sabouraud dextrose agar and their staining patterns, they were considered to be fungal-like organisms. On the other hand, formation of endospores and the presence of non-chitin rigid cell walls indicated that *Prototheca sp*. might belong to algae. Currently, based on molecular analysis, they are classified as colorless green algae, closely related to *Chlorella* sp. They lack genes for photosynthesis and therefore can use organic carbon sources in almost every environment, including fossil oils (Walker et al., 1975; Pore et al., 1983). The cell walls of these microalgae contain sporopollenin, a robust biopolymer, rendering them highly resistant to mechanical stress, physical, and chemical treatment and to enzymatic degradation (Lloyd and Turner, 1968; Ueno, 2009). Currently there are six generally accepted *Prototheca* species: *P. ulmea, P. stagnora, P. cutis, P. wickerhamii, P. blaschkeae*, and *P. zopfii* (Roesler et al., 2006; Satoh et al., 2010). At present *P. zopfii* is subdivided in two genotypes (GT), GT1 and GT2. Although termed a “genus,” *Prototheca* seems to be paraphyletic. *P. wickerhamii* is more closely related to *Auxenachlorella* sp. than to the other *Prototheca* species (Ueno et al., 2003, 2005). *P. wickerhamii, P cutis, P. zopfii*, and *P. blaschkeae* are reported to cause infections in vertebrates, primarily mammals like household pets (cats and dogs), livestock (pigs and cattle) and also humans (Pore and Shahan, 1988; Leimann et al., 2004; Camboim et al., 2011). Interestingly *P. zopfii* GT2 is associated with the most severe forms of protothecosis like bovine mastitis or encephalitis of dogs, while GT1 is considered to be non-pathogenic (Möller et al., 2007; Osumi et al., 2008; Kishimoto et al., 2010). *P. blaschkeae* (previously *P. zopfii* GT3), is found to be associated with subclinical bovine mastitis and it is isolated much less frequently than *P. zopfii* GT2 (Marques et al., 2008; Jagielski et al., 2011). Only one case of severe systemic infection due to *P. blaschkeae* has been reported so far (Thompson et al., 2009).

The disease condition is referred as protothecosis, which displays varying clinical patterns depending on the host species. In human, local cutaneous lesions, infections of the olecranon bursa and disseminated infection are observed (Lass-Florl and Mayr, 2007). In cattle, bovine mastitis represents the predominant manifestation of protothecosis. Acute infections result in granulomatous mastitis, whereas chronic progression cases are associated with decreasing milk yield and increasing cell numbers (Lerche, 1954). *Prototheca* also represent a potential zoonotic risk as they persist after pasteurization of milk due to their heat-resistant nature (Melville et al., 1999).

Dogs typically suffer from disseminated infections usually beginning with chronic bloody diarrhea followed by neurologic symptoms like ataxia, blindness, deafness, or seizure (Stenner et al., 2007; Ribeiro et al., 2009). The majority of cases occur in female dogs, mostly in boxers, collies, and giant schnauzers. The outcome of an infection is usually fatal (Stenner et al., 2007). As in cattle, most frequently *P. zopfii* GT2 is isolated from canine protothecosis (Font et al., 2014).

Little is known about the pathogenesis in humans, however immunosuppression and drug abuse have been identified as possible risk factors (Chao et al., 2002). Therapy options are limited in general. While local infections can be removed by surgery, disseminated infections in humans are treated experimentally with diverse mixtures of antimycotics and antibiotics with varying success (Thiele and Bergmann, 2002; Zhao et al., 2004).

The course of protothecosis and the difference in pathogenicity between the different *Prototheca* species and genotypes are poorly understood. So far no virulence factors are known and the severe inflammatory reactions in bovine mammary glands are reminiscent of toxin activity. Earlier attempts to identify possible antigens using rabbits experimentally immunized with living *Prototheca* cells remain inconclusive due to the fact that natural *Prototheca* infection among rabbits has not been described yet (Irrgang et al., 2015). Therefore, the aim of this study was to identify immunogenic proteins using the antibodies present in the sera of dogs which were naturally infected with *P. zopfii* GT2. Additionally, cross-reactivity of the sera was determined by western blot analysis of *P. zopfii* GT1, *P. zopfii* GT2, and *P. blaschkeae*.

## Materials and methods

### Dogs and sera

Sera from three dogs naturally infected with Prototheca collected during the routine diagnosis at the Institute of Animal Health and Environmental Hygiene, Freie Universität Berlin, were utilized in this study.

Case 1 (serum P): A female giant schnauzer (age: 7 years) suffered from chronic therapy-resistant diarrhea for more than one and a half year followed by a severe systemic protothecosis with neurological symptoms. Serum samples were obtained during the course of infection and pooled before use.
Case 2 (serum L): The female crossbreed (age: 9 years) suffered from a severe systemic protothecosis.
Case 3 (serum B): A female giant schnauzer with systemic protothecosis showing severe neurological symptoms.

Despite intensive therapeutic measures all three dogs finally had to be euthanized. *Prototheca* was isolated from the first two cases (P and L) and identified as *P. zopfii* GT2 using recommended molecular analysis (Möller et al., 2007) and MALDI TOF MS (Murugaiyan et al., 2012). *Prototheca* was not isolated from the third case; however, in addition to signs and symptoms, histological analysis, and species-specific PCR revealed the infection. Hence, the third sample (serum B) was utilized for comparative studies.

Sera (C, R, A) from three healthy dogs with no history of symptoms were used as negative controls.

### Strains

The following type strains of different species or genotypes from the culture collection of the Institute of Animal Health and Environmental Hygiene were used: *P. zopfii* GT1 (SAG 2063^T^) (Roesler et al., 2003), *P. zopfii* GT2 (SAG 2021^T^) (Roesler et al., 2001), and *P. blaschkeae* (SAG 2064^T^) (Roesler et al., 2006). These strains were originally isolated by the investigators and then deposited at the public strain collection “Stammsammlung für Algenkulturen” of the University of Göttingen (SAG). Additionally, the strains isolated from the dogs, cases 1 and 2, were designated as PZ-L and PZ-P, respectively.

### Culture conditions and protein extraction

Strains were cultured on Sabouraud dextrose agar at 37°C (except SAG 2063^T^, which was cultured at 28°C) for 48 h. The culture temperature was chosen to represent the original environmental conditions and from our previous experience (Murugaiyan et al., 2012). A loop of colony was transferred in Sabouraud dextrose liquid medium and overnight cultured by shaking at 135 rpm. Following which, 2 ml of this culture was inoculated in 200 ml of medium and cultured as above. The whole-cell protein extraction was carried out as described (Murugaiyan et al., 2013). In brief, cells were harvested from 15 ml of culture by centrifugation at 2000 g for 5 min at room temperature. The pellet was washed two times in 1.5 ml PBS and resuspended in 1 ml lysis buffer (20 mM HEPES, pH 7.4; 1 mM EDTA with 1% protease inhibitor cocktail tablet, 1% Triton X and 10% glycerol). Cell lysis was assisted by an additional sonication step on ice. After subsequent centrifugation, supernatant was collected and protein concentration was determined with Quick Start Bradford 1x Reagent Dye (BioRad, Munich, Germany).

### Gel electrophoresis

For sodium dodecyl sulfate polyacrylamide gel electrophoresis (1DE) 150 μg whole cell protein of SAG 2021^T^ was separated using a 12% SDS gel with preparative comb (Laemmli, 1970). The separation was carried out with 10 mA per gel for 15 min followed by increasing the current to 15 mA per gel until the dye reached the bottom of the gel.

Two-dimensional electrophoresis (2DE) was performed with 250 μg (western blot analysis) and with 500 μg up to 1 mg (preparative gel for protein identification). All the reagents and accessories for isoelectric focussing (IEF) were purchased from GE Healthcare. After acetone precipitation of the proteins, the pellet was resuspended in DeStreak Rehydration Solution containing 0.5% IPG buffer, centrifuged for 20 min at 18500 g, supernatant was transferred to 7 cm immobilized pH gradient (IPG) drystrips (pH 3–10) and covered with mineral oil for passive rehydration overnight. The manufacturer's recommendations on IEF was followed: 300 V for 2 h, 300–1000 V for 1 h, 1000–5000 V for 2 h, holding 5000 V for another 1 h 30 min, total of 14750 V/h with current of 50 μA per strip. Subsequently, strips were incubated for 15 min for reduction in 2% dithioerythritol in equilibration buffer (0.05 M trichloroethylene HCl pH 8.8, 6 M urea, 30% glycerol, 4% SDS, and 0.002% bromophenol blue). Strips were then rinsed with distilled water and subjected to alkylation with 2.5% iodoacetamide in the equilibration buffer for 15 min. Strips were stored at -20°C until further use. The second dimension separation was carried out on 12% SDS polyacrylamide gel using same conditions described for 1DE.

Prestained protein ladder (PageRuler™Plus, ThermoScientific, Rockford, USA) was used as marker for 1DE as well as for 2DE. If desired the gels were stained with Colloidal Coomassie Brilliant Blue (Candiano et al., 2004).

### Western blot

Following protein separation on 1DE or 2DE, proteins were transferred to nitrocellulose membrane (BioRad, Munich, Germany) in a semi-dry blotting chamber at 80 mA per gel for 90 min using standard Towbin Buffer (Towbin et al., 1979). Membranes were blocked by incubating them overnight in 1% skin milk in Tris-buffered saline (TBS, 20 mM Tris, 500 mM NaCl pH 7.5), followed by three washing steps in TBS for 10 min. Membranes were stored at −20°C until further use. To facilitate simultaneous incubation of nine different serum samples 1D blot membrane, incubation manifolds (PR150, Hoefer, Holliston, USA) was utilized. 1DE and 2DE membranes were incubated with serum (1:100 in TBS with 0.5% Tween 20 referred as TBST) for 90 min. The membrane was then washed twice each for 10 min with TBS and then incubated with Anti-dog IgG (Bethyl Laboratories, Montgomery, USA), 1:1500 diluted, for 90 min. Following secondary antibody exposure the membrane was washed twice 10 min each with TBTS and finally washed for 5 min with TBS. Visualization was carried out by using tetramethylbenzidine (TMB, Sigma-Aldrich, Germany) following the manufacturer's instructions. The two serum samples (L and P) were applied to all five strains used in this study (Table 1). After stripping one membrane for each strain that had previously been developed with one of the positive sera, the negative serum C was applied. Membrane stripping was carried out by 30 min incubation with stripping buffer (62.5 mM Tris pH 6.7, 2% SDS, and 100 mM 2-mercaptoethanol). Then the membranes were washed several times in purified water followed by blocking overnight and proceeding with second antibody treatment.

**Table 1:** **Overview of performed western blots and sera used**.

**Cell lysate/WB**	**SAG 2063^T^ (*P. zopfii* GT1)**	**PZ-P (*P. zopfii* GT2)**	**SAG 2021^T^(*P. zopfii* GT2)**	**PZ-L (*P. zopfii* GT2)**	**SAG 2064^T^ (*P. blaschkeae*)**
Serum	L (+)	L (+)	L (+)	L (+)	L (+)
	P (+)	P (+)	P (+)	P (+)	P (+)
	C (−)	C (−)	C (−)	C (−)	C (−)

*+, serum of infection with P. zopfii; P, serum of dog 1 infected with strain PZ-P; L, serum of dog 2 infected with strain PZ-L; −, negative control (= serum “C”); GT, genotype*.

2DE western blots were analyzed and compared using Delta2D software version 4.5.0 (Decodon, Greifswald, Germany) and the corresponding gel spots were identified on the Coomassie-stained gel by overlaying the western blot image with the image of the 2D gel.

### Trypsin digestion and protein identification

Protein spots that matched with the corresponding signals of the western blot were excised. The spots were subjected to trypsin digestion as described (Wareth et al., 2015). Thereafter 5 μl of organic solvent (OS) (33% of acetonitrile in 0.1% trifluoroacetic acid) was added to the samples. One microliter of the peptide solution was mixed well with 1 μl of α-Cyano-4-hydroxy-cinnamic acid (HCCA; 15 μg/μl in OS), spotted on a ground steel MTP 384 target plate, air-dried completely and then measurements were carried out using matrix-assisted laser desorption ionization with a time-of-flight mass spectrometer (MALDI-TOF MS) (Ultraflex II TOF/TOF, Bruker Daltonics, Bremen, Germany). Peptide Calibration Standard II (Bruker Daltonics, Bremen, Germany) that covers the range between 700 and 3500 Da was used for calibration, MALDI TOF peptide mass finger print (PMF) spectra were acquired in positive reflection mode with the following setting, ion source 1: 25 kV, ion source 2: 21.60 kV, lens: 10.50 kV, reflector 1: 26.30 kV, reflector 2: 13.60 kV. Measurements were carried out in the m/z range of 1000–3500 and seven of the intensive peaks were picked for lift spectra. The MS/MS product ion spectra were recorded in the laser-induced dissociation mode with the following settings; ion source 1: 8 kV, ion source 2: 7.20 kV, lens: 3.60 kV, reflector 1: 29.50 kV, reflector 2: 13.90 kV, lift 1: 19.00 kV, lift 2: 3.00 kV. Subsequently, BioTools 3.0 (Bruker Daltonics, Bremen, Germany) was utilized to compare MS/MS data using MASCOT, www.matrixscience.com) against all entries of NCBInr (GenBank) with the following parameters: trypsin digestion- up to one missed cleavage; fixed modifications-carbamidomethyl (C); variable modifications -oxidation (M); peptide tol.: ±100 ppm; MS/MS tol.: ±0.8 Da and peptide charge:+1.

Furthermore, seven samples were additionally analyzed by Proteome Factory (Berlin, Germany) using nanoLC–ESI-MSMS and the database search was carried out as described above.

### Flow cytometry

Expression of GAPDH on the surface of SAG 2063, SAG 2021, and SAG 2064 cells was analyzed by flow cytometry. A total of 1 × 10^7^ cells (overnight culture as described above) were fixed in 4% paraformaldehyde for 15 min, followed by one washing step with phosphate buffered saline (PBS). Cells were incubated with 5–10 μg/ml polyclonal anti-GAPDH (Rockland, Limerick, USA) for 1 h at 37°C. After two additional washing steps, cells were stained with Alexa-Fluor 488-labeled goat anti-rabbit IgG (1/500 dilution) for 1 h. After final two washes, cells were analyzed in FACSCalibur™ flow cytometer (BD Bioscience, Erembodegem, Belgium). The intensity of fluorescence was analyzed using Flowing software (University of Turku, Finland). The experiment was repeated three times independently.

## Results

### Visual analysis of western blots

In 2D-PAGE, proteins from *Prototheca* whole-cell extracts were evenly distributed over the selected pI range and well-resolved, as shown in Supplementary Material (Figure S1). Comparing the infectious strains PZ-L and PZ-P show high similarities between them and also each of them with SAG 2021^T^, supporting their assignment as *P. zopfii* GT2 also at the proteome level.

**Figure 1:**
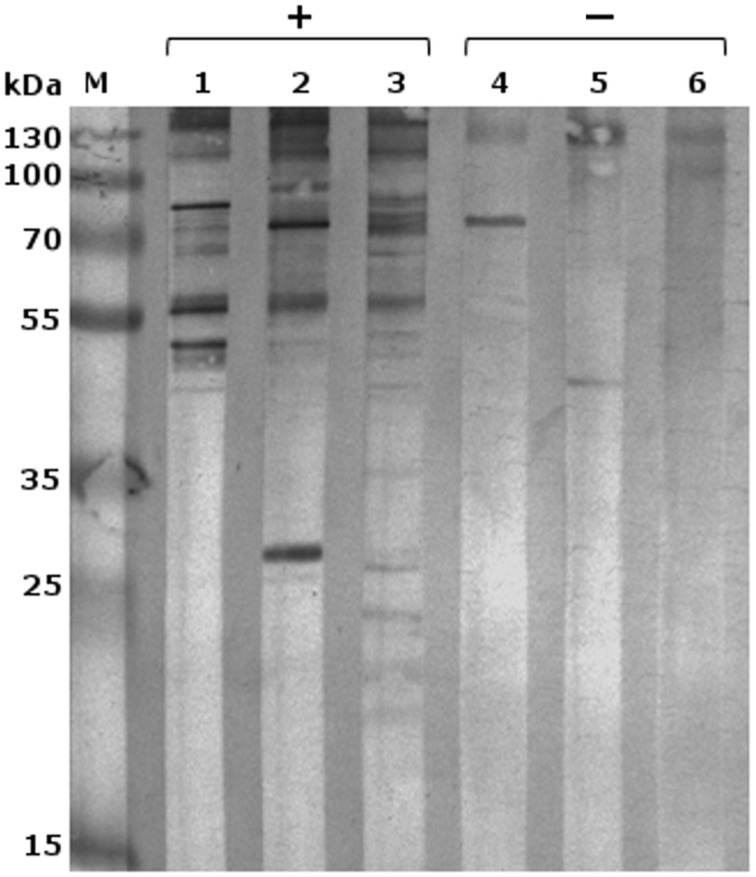
**1DE-western blot of *P. zopfii* GT2 (SAG 2021) with different immune sera; M, marker; lanes 1–3 sera from infected dogs; and lanes 4–6 negative controls; 1, serum L; 2, serum P; 3, serum B; 4, serum R; 5, serum C; 6, serum A**.

**Figure 2:**
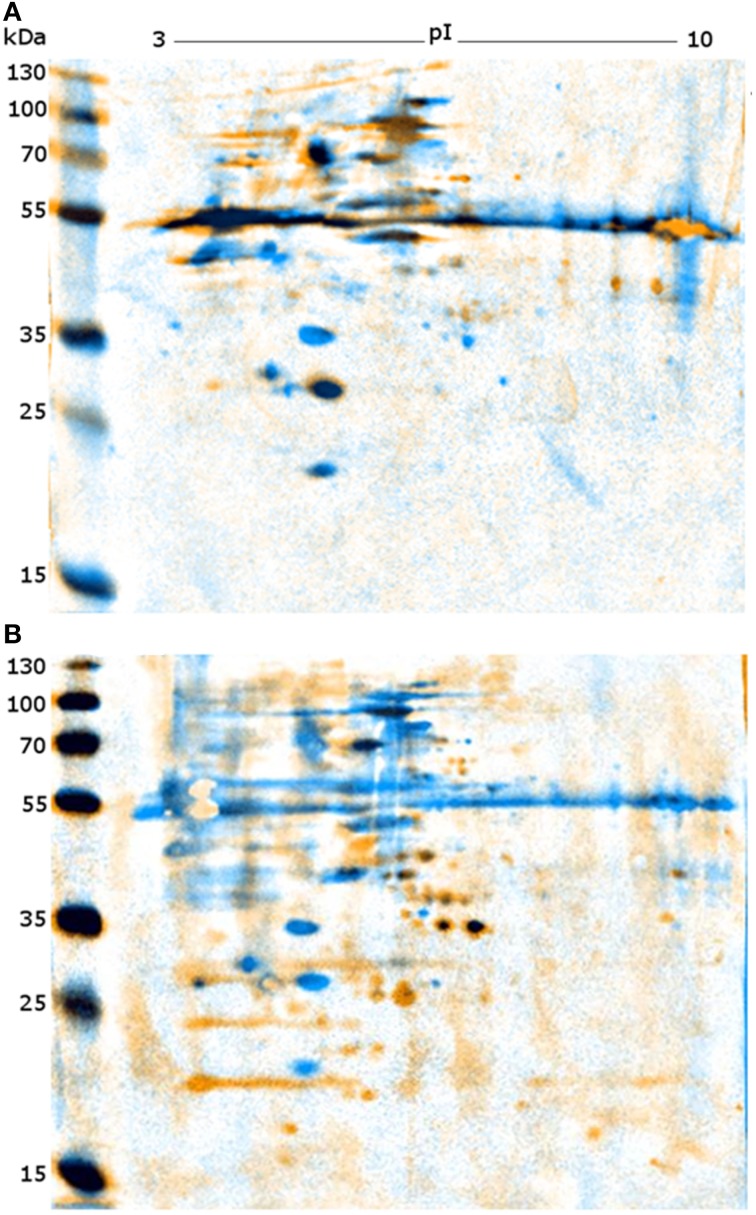
**Representative images of overlaid 2DE-western blots. (A)** Western blots of PZ-L (blue) and SAG 2063 (yellow) each incubated with serum L. **(B)** Western blots of SAG 2021 incubated with serum L (blue) and serum P (yellow).

Following optimization of serum and secondary antibody concentrations, all positive and negative serum samples were compared on a 1DE western blot membrane of SAG 2021^T^ to obtain an overview (Figure 1). All samples differed substantially in the resulting signal pattern which highlights the immunological variations among individuals. These differences were also reflected in their respective 2DE patterns. These were compared by pairwise overlays using image analysis software (Delta2D). Comparing the 2DE western blot patterns obtained by one of the serum samples on different cell lysates resulted in a high strain-, genotype- and species-cross-reactivity of both sera (Figure 2A). This suggest that antigen epitopes do not vary much between the *Prototheca* species and genotypes even though small differences are visible.

Comparison of both sera on a single membrane enables the identification of distinct protein spots that originate from individual immune responses (Figure 2B). Altogether, a total of 227 proteins spots were detected from five membranes. For a better understanding of the large set of signal information across different membranes, the signals were classified in six categories based on visual comparison (Table 2).

**Table 2:** **Categorization of the western blot signals based on visual comparison after overlaying the blots obtained from the strains and sera used in this study**.

**Category**	**Name**	**Characterization**	**No. of proteins (L/P)**
I	Individual antigen	Signal of a serum on corresponding western blot membrane	11 (8/3)
II	genotype-specific antigen	Signals only detected on western blots of *P. zopfii* GT2	19 (6/13)
III	Common antigen	Signals detectable on all membranes of investigated strains	29 (17/12)
IV	Pathogenic-specific^*^	Signals for *P. zopfii* GT2 and *P. blaschkeae*	10 (8/2)
V	Species-specific antigen	Signals for *P. zopfii* (GT1+2)	12 (7/5)
VI	Unspecific antigen	Unspecific binding of antigens	146 (58/88)
			∑ 227 (104/123)

* *This term is based on species included in the present study—the other known infectious species P. wickerhamii or P. cutis were not included; GT, genotype; P, serum of dog 1 infected with strain PZ-P; L, serum of dog 2 infected with strain PZ-L*.

**Figure 3:**
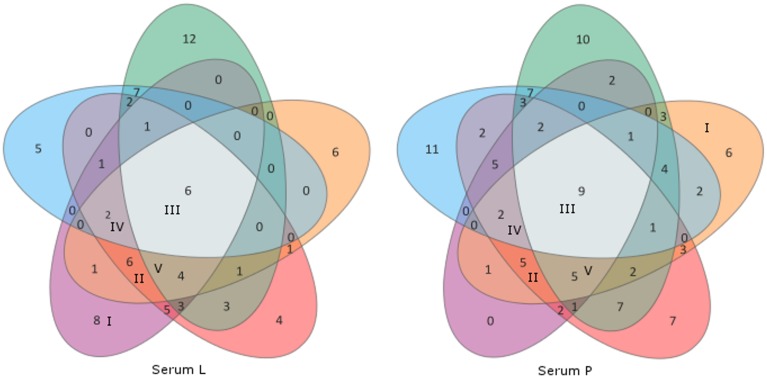
**Venn diagrams of the sera L and P showing distribution of cross-reactivity of each serum**. Western blot membranes: green, SAG 2063 (*P. zopfii* GT1); orange, PZ-P; red, SAG 2021; violet, PZ-L (all *P. zopfii* GT2); blue, SAG 2064 (*P. blaschkeae*). Associated categories I–V (Table 2) are given in Roman numerals in the respective fields, all remaining unlabeled fields belong to category VI.

Categories I–V consist of homologous signals which means that the signal can also be obtained on the associated western blot membrane (PZ-L and serum L; PZ-P and serum P). Figure 3 depicts the distribution of the signals in comparison to the sera used in this study. Serum L detects six and serum P nine proteins on western blot membranes of all five strains. Of note, the data of Figure 3 and Table 2 do not have to correspond. The Venn diagram only incorporates the events of antigen recognition so similar signals are not counted repeatedly, whereas data in Table 2 documents every labeled spot. According to Table 2, the major part of the western blot signals originated from non-specific antibody binding (category VI). Although proteins of special interest would fall into category II and ideally be detected by both immune sera, all proteins have been analyzed. A preparative 2DE gel loaded with a higher amount of proteins (1 mg) was performed so as to enable efficient identification of proteins. In total, 198 proteins have been excised and analyzed via MALDI-TOF MS.

### Immunoreactive proteins

In summary 86 out of these 198 proteins could be identified. Most of the identified proteins appear to be involved in general cellular processes such as metabolism, cell cycle or, gene expression.

A structured overview is given in Table 3 where proteins are listed, which are either of special interest or represent typical results. Results obtained repeatedly are listed only once. The overall results, including spot numbers, hypothetical proteins, or implausible assignments are available in the Supplementary Material (Table S1).

**Table 3:** **Representative list of identified proteins by MALDI TOF MS**.

**Protein**	**Organism**	**MOWSE Score**	**Category**	**Serum**	**Function**	**Identified from multiple gels**
Glyceraldehyde-3-phosphate dehydrogenase	*Acidobacteria bacterium*	247^*^	III	L/P	Glycolysis	+
Heat shock protein 70	*Chlorella variabilis*	164	II	L/P	Intracellular: part of chaperon system; stress response Extracellular: proinflammatory immune response	+
Translation elongation factor- like protein (EF-1α)	*Parachlorella kessleri*	159	III	L/P	Protein biosynthesis	+^a^
ATPase alpha subunit	*Passiflora suberosa*	132	II	L	Energy metabolism	+^a^
Triosephosphate isomerase	*Fusobacterium nucleatum*	110	III	P	Glycolysis	^a^^,^ ^b^
Malate dehydrogenase	*Leishmania infantum*	115	II	L/P	diverse metabolic pathways	+^a^^,^ ^b^
PREDICTED: glutamyl-tRNA(Gln) amidotransferase subunit B, mitochondrial	*Pelodiscus sinensis*	102	VI	P	Translation	–
Phenylalanyl-tRNA synthetase beta subunit	*Archaeoglobus sulfaticallidus*	101	II	L	Translation, ubiquitous	–
LysR family transcriptional regulator	*Pseudomonas psychrotolerans*	99	III	L	Prokaryotic transcriptional regulator i.e., virulence	–
PREDICTED: protein BMH2 isoform 1	*Strongylocentrotus purpuratus*	94^**^	III	P	Member of 14-3-3 protein family–> cell signaling	–
Radical SAM protein, TIGR01212	*Sorangium cellulosum*	93	V	L/P	Enzyme super family, catalytic metabolism	–
Putative phosphomannomutase	*Listeria ivanovii* subsp. ivanovii	91	VI	L/P	Protein gylcosylation, mannose synthesis	–
Protocatechuate 4,5-dioxygenase subunit alpha	*Hydrogenophaga* sp.	90^**^	III	P	PCA –> break-down of lignin	–
3-dehydroquinate synthase	*Streptomyces rimosus*	89	I	P	Enzyme of shikimate-pathway	–

*The MOWSE score (MOlecular Weight SEarch score) is calculated by −10 log (P), where P is the probability that the observed match is a random event. The identification is considered to be valid if the MOWSE score is greater than or equal to the significance threshold (P < 0.05). Category based on signals of different blots out of strains and sera used. P, serum of dog 1 infected with strain PZ-P; L, serum of dog 2 infected with strain PZ-L*.

* *Score from additional validation using nanoLC–ESI-MSMS*.

** *Settings of MS/MS tolerance changed to 1.2 Da*.

a *Identification values from earlier study (Irrgang et al., 2015)*.

b *Results as reported in Murugaiyan et al. (2013)*.

One protein that was identified clearly and repeatedly is heat shock protein 70 (Hsp70). It was also identified from culture supernatant (unpublished data). Hsp70 was classified as genotype-specific antigen and traceable by both immune systems. Another example is glyceraldehyde-3-phosphate dehydrogenase (GAPDH), but in contrast to Hsp70 GADPH is classified as a common antigen with stronger signals obtained using serum P. Interestingly serum P reacts more intensively on the membrane of PZ-L than on its homologous membrane of PZ-P. Triosephosphate isomerase could be detected by serum L on all membranes. Although identification via MALDI-TOF MS was not successful in this study, the protein identification was known from our earlier studies of difference gel electrophoresis (DIGE) and experimentally infected rabbits (Murugaiyan et al., 2013; Irrgang et al., 2015). Other recurrently detected proteins include malate dehydrogenase, elongation factor 1alpha (EF-1α) and ATPase. ATPase, phenylalanyl-tRNA synthetase, Hsp70, and malate dehydrogenase represent antigens specific for genotype 2 of *P. zopfii*.

### Flow cytometry analysis

**Figure 4:**
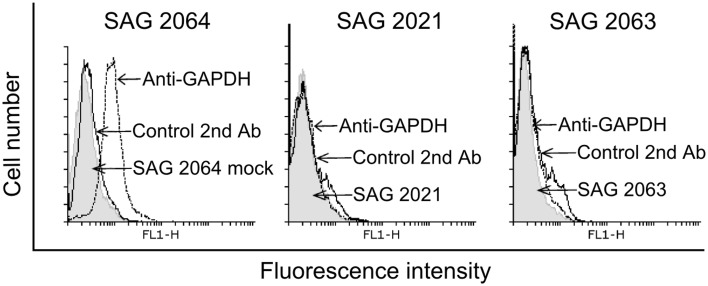
**Flow cytometry analyses for GAPDH expression on the surface of *P. blaschkeae* (SAG 2064), *P. zopfii GT2* (SAG 2021), and *P. zopfii GT1* (SAG 2063)**. A representative image is shown. Cells in suspension were incubated with anti-GAPDH polyclonal antibodies, followed by incubation with Alexa-Fluor 488-labeled goat anti-rabbit IgG. As controls, cells were incubated only with the Alexa-Fluor 488-labeled goat anti-rabbit IgG to exclude any background. The experiment was repeated three independent times and results of one representative experiment are shown.

To examine whether GAPDH is expressed on the surface of *Prototheca*, a flow-cytometry analysis was performed (Figure 4). In this experiment, GADPH was detected on the surface of *P. blaschkeae* (in 20% of the cell population) but not of *P. zopfii* (none of the genotypes).

## Discussion

The microalgae *Prototheca* spp. are non-colored relatives of the well-known green algae, *Chlorella* sp. Their unique ability as a plant to cause severe and difficult-to-treat infections in vertebrates makes them fascinating for research. The mechanism of pathogenesis and their differences in pathogenicity are not understood yet, but valid knowledge hereof would be the key to develop tools for therapy (antimicrobial drugs) or for prevention by immunization. One aspect is the identification of immunoreactive proteins of *Prototheca* cells targeted by the present study. For that purpose sera from two dogs suffering from protothecosis caused by *P. zopfii* GT2 were used for western blot studies and MALDI-TOF MS-based identification of immunodominant proteins. The investigations involved strains of *P. zopfii* GT1 as non-pathogenic agent, *P. zopfii* GT2 as pathogenic representative with high prevalence and clinical manifestations and *P. blaschkeae* as pathogen with subclinical manifestations in cattle. Phylogenetic studies revealed a close relationship with distinct clusters of the species and genotypes (Ueno et al., 2003, 2005; Roesler et al., 2006). *P. wickerhamii*, another pathogenic *Prototheca* species is related much more closely to *Auxenachlorella* sp. than to the other *Prototheca* species and was therefore excluded from the present study.

The western blot signals were accordingly classified into six categories for the species included in this study. Genotype-specific antigens for *P. zopfii* GT 2 (category II), which were detected in both serum samples, were focused on since they might indicate virulence factors. With regard to bovine mastitis, caused by *P. zopfii* GT2 *or P. blaschkeae*, immunoreactive proteins assigned to category IV might be also of special interest. However, none of these proteins could be identified. The relatively low identification rate for *Prototheca* proteins is due to the fact that information on algal proteins is scarce since algae are strongly under-represented in the protein sequence databases. As a consequence of the absence of *Prototheca*-specific information in the public repositories only homologous proteins from other organisms can be retrieved and the identification scores generally tend to be lower. Regardless of this complication 86 proteins could be identified with significant scores. Due to the lack of *Prototheca* sequences in the database, most of the identified proteins are from other organisms. The fact that identifications are from very diverse species indicates that actually the proteins are from *Prototheca* rather than from one particular contaminating source, in which case more proteins should be identified from this contaminating species. Many of the identified proteins were metabolic and housekeeping proteins which might possess peptide sequences that are well conserved across species. We believe that in the case of “orphan species” such as *Prototheca* only those proteins are identified whose sequences are relatively well conserved across the species, while possibly more *Prototheca*-specific factors, where sequence divergence is larger, might be lost.

Hsp70 which was repeatedly identified in *P. zopfii* GT2 with both sera utilized, appears to be the major antigen of *Prototheca* infections. In our previous investigation using sera from experimentally infected rabbits Hsp70 was identified as immunoreactive, too (Irrgang et al., 2015). Hsp70 is a highly conserved protein family some members of which are stress-inducible proteins with chaperone functions while others are stress-independent proteins with house-keeping functions (Daugaard et al., 2007). Despite their mainly intracellular functions, Hsp70 also may act as cytokine when located extracellularly (Asea et al., 2000). Hence, these proteins are known to be immunogenic in several eukaryotic infections. Hsp70 represents the main target of immune response against *Crypotcocoocus neoformans* (Kakeya et al., 1997, 1999) and might be a target for immunization (Chaturvedi et al., 2013). Hsp70 of *Schistosoma mansoni* was found to be immunoreactive in humans (Ludolf et al., 2014) and Hsp70 of the nematode *Anisakis* was considered to be an allergenic protein (Arcos et al., 2014). Eliciting a strong pro-inflammatory response by Hsp70 of *Prototheca zopfii* GT2 might be an explanation for the observed inflammation especially of the udder of infected dairy cattle. This is consistent with the fact that the uptake rate of *P. zopfii* GT2 by macrophages is higher in comparison to GT1 going along with higher resistance to digestion (unpublished data). Furthermore, Hsp70 was also detected in culture supernatants of *Prototheca*. This supports the theory of an immunogenic role of Hsp70 during infection.

Like Hsp70, glyceraldehyde-3-phopshate dehydrogenase (GAPDH) was found to be an allergen of *Anisakis* and also an immunogenic protein of *Schistosoma* and *Cryptococcus* (Martins et al., 2013; Arcos et al., 2014; Ludolf et al., 2014). Additionally there are several studies which evince the presence of house-keeping enzymes such as GAPDH or triosephosphate isomerase at the surface of pathogens (Pancholi and Chhatwal, 2003). There, GAPDH acts as virulence factor due to adherence on host cells (Fu, 2013). Moreover, surface GAPDH of group A streptococci is an ADP-ribosylating enzyme, with ADP ribosylation being the mechanism of some bacterial toxins (Moss and Vaughan, 1988; Pancholi and Fischetti, 1993). Unfortunately, there is little known about surface proteins of *Prototheca* cells. In order to verify the presence of GAPDH on the *Prototheca* surface, the cells were subjected to a flow cytometry analysis, the results of which indicated the presence of GAPDH on the surface of *P. blaschkeae* but not of *P. zopfii*. This might be one of the reasons for varying mechanism in pathogenesis between those species. Besides GADPH, the other identified enzymes such as triosephosphate isomerase and enolase have also been described to be present on the surface of pathogens. Hence, further research to confirm the presence of these enzymes on the surface of pathogenic *Prototheca* sp. is highly recommended.

Phosphomannomutase is another protein of immunological interest. This enzyme is supposed to be an indirect virulence factor for *Cryptococcus* sp. and was detected through immunoblotting using patient sera (Martins et al., 2013). It is needed for synthesizing mannose, a major component of the *Cryptococcus* capsule which represents the main virulence factor. The capsule protects the cells against immune system, either because of being an antiphagocytic factor or due to having immunosuppressive effects when extruded into surrounded tissue and liquids. Mannose is a component of lipopolysaccharide (LPS), which supports the earlier description of LPS-like molecules in the cell wall of *Prototheca* (Bedick et al., 2001). This aLPS was able to stimulate the insect immune system but was not recognized by murine macrophages. Potential immunosuppressive effects of intact *Prototheca wickerhamii* cells were also reported by Perez et al., which is in contrast to possible immune activation by Hsp70 (Pérez et al., 1997). The sera showed no cross-reactivity, however, with Hsp70 of the non-pathogenic genotype 1 of *P. zopfii* or of *P. blaschkeae*, indicating a special form or genotype 2-specific epitopes of Hsp70. In contrast the sera were only reactive with phosphomannomutase of SAG 2063 (*P. zopfii* GT1) and therefore with the non-pathogenic form. In case *P. zopfii* GT1 might additionally lack a key virulence factor, this could be an explanation for their non-pathogenicity. Furthermore, Pérez et al. (1997) proposed that only dead *Prototheca* cells might induce an inflammatory response. This is supported by the results of the present study, where predominantly intracellularly located antigens were identified besides the discussed exceptions.

One of these intracellularly located antigens is malate dehydrogenase (MDH). This key enzyme of many metabolic pathways, including citric acid cycle, seems to play a specific role in antigen recognition of eukaryotic infections, especially fungal infections. The sera of patients with aspergillosis, infections of *Candida albicans*, or *Paracoccidioides brasiliensis* were also reported to possess antibodies against MDH (da Fonseca et al., 2001; Pitarch et al., 2004; Shi et al., 2012). The influence of MDH on inducing a host immune response or its role in pathogenicity is not clear. This enzyme appeared to be one of the antigens specific for genotype 2 of *P. zopfii*.

Taken together this study reveals among the immunodominant proteins of *Prototheca* a certain number of proteins that are also known as eukaryotic antigens. Protothecosis is a rare but severe infection with an increasing economic impact in dairy farming. Since there are no available therapies, options of immunization or immune therapy should be a focus of *Prototheca* research. Protein extracts might be used for inducing a protective immune response as a first approach. Once the genome of *Prototheca* is sequenced, *Prototheca*-specific pathways of infection could be clarified and a specific immunization based on recombinant proteins may become possible.

## Conflict of interest statement

The authors declare that the research was conducted in the absence of any commercial or financial relationships that could be construed as a potential conflict of interest.

## Supplementary material

Figure S1 **2D gel electrophoretic pattern of *Prototheca* strains**. Spot ID with prefix L and P indicates that the western blot signals were observed with serum L and P, respectively. **(A)** SAG 2021 (*P. zopfii* GT2), **(B)** SAG 2063 (*P. zopfii* GT1), **(C)** SAG 2064 (*P. blaschkeae*), **(D)** PZ-L (*P. zopfii* GT2), and **(E)** PZ-P (*P. zopfii* GT2).

Table S1 **List of proteins identified by MALDI TOF MS**. Spot ID is the spot excised based on the western blot signals where L and P represents the signals out of serum L and P, respectively and K represents the signals obtained from both of the sera with results from earlier study. Gel positions of spot numbers are shown in Supplementary Figure S1. Gel represents 2D gel from where spots were excised, *P. zopfii* GT1 (SAG 2063), *P. zopfii* GT2 (SAG SAG 2021, PZ-L, PZ-P), and *P. blaschkeae* (SAG 2064). NCBI Acc. No. is the accession number of the proteins identified by comparing the peptide sequence with NCBI databank, Uniprot Acc. No. is the accession number of the proteins with Uniprot. MW, molecular weight calculated from identified protein sequence, The MOWSE score (MOlecular Weight SEarch score) is calculated by −10 log (*P*), where *P* is the probability that the observed match is a random event. The identification is considered to be valid if the MOWSE score is greater than or equal to the significance threshold (*P* < 0.05). This list includes only those proteins identified with a, pI, isoelectric point as calculated from the identified protein sequence, sequence coverage is the % of sequence of the matching peptides and category represents the grouping of western blotting signal (I-Individual antigen, II-genotype-specific antigen, III-common antigen, IV-Pathogenic specific for the species included in this study, V-species specific antigen, and VI-Unspecified antigen). **(A)** MS/MS product ion search result and **(B)** MS (PMF) ion search result.
