# Supplementary figures and images for: Well-known surface and extracellular antigens of pathogenic microorganisms among the immunodominant proteins of the infectious microalgae Prototheca zopfii
Source: Front Cell Infect Microbiol. 2015 Sep 29;5:67. doi: 10.3389/fcimb.2015.00067 (PMC4586511; doi:10.3389/fcimb.2015.00067)

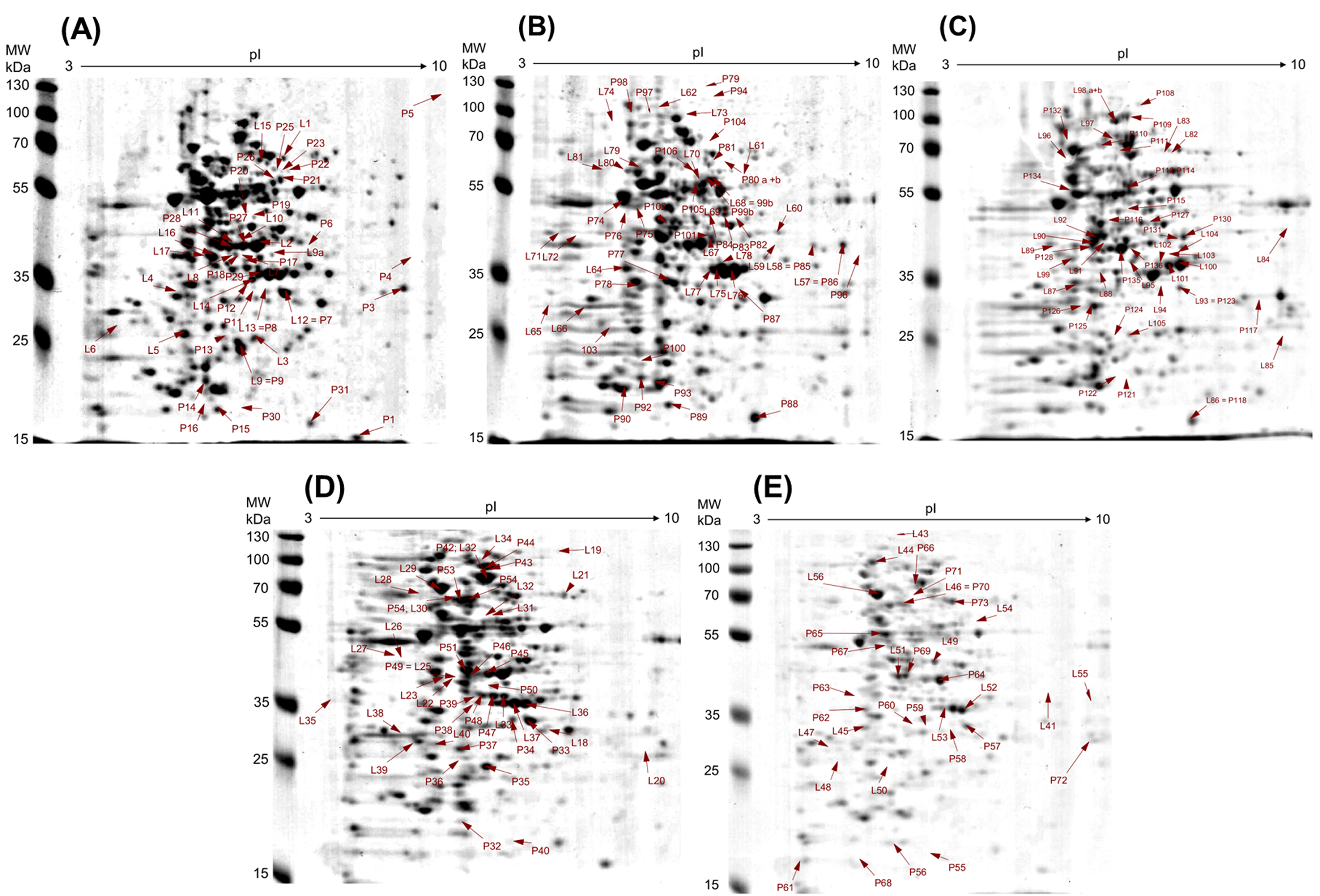

Supplement: Figure S1 — 2D gel electrophoretic pattern of Prototheca strains. Spot ID with prefix L and P indicates that the western blot signals were observed with serum L and P, respectively. (A) SAG 2021 (P. zopfii GT2), (B) SAG 2063 (P. zopfii GT1), (C) SAG 2064 (P. blaschkeae), (D) PZ-L (P. zopfii GT2), and (E) PZ-P (P. zopfii GT2). [file Image1.TIFF]
