# Supplementary material for: Well-known surface and extracellular antigens of pathogenic microorganisms among the immunodominant proteins of the infectious microalgae Prototheca zopfii
Source: Front Cell Infect Microbiol. 2015 Sep 29;5:67. doi: 10.3389/fcimb.2015.00067 (PMC4586511; doi:10.3389/fcimb.2015.00067)
Supplement: Table S1 — List of proteins identified by MALDI TOF MS. Spot ID is the spot excised based on the western blot signals where L and P represents the signals out of serum L and P, respectively and K represents the signals obtained from both of the sera with results from earlier study. Gel positions of spot numbers are shown in Supplementary Figure S1. Gel represents 2D gel from where spots were excised, P. zopfii GT1 (SAG 2063), P. zopfii GT2 (SAG SAG 2021, PZ-L, PZ-P), and P. blaschkeae (SAG 2064). NCBI Acc. No. is the accession number of the proteins identified by comparing the peptide sequence with NCBI databank, Uniprot Acc. No. is the accession number of the proteins with Uniprot. MW, molecular weight calculated from identified protein sequence, The MOWSE score (MOlecular Weight SEarch score) is calculated by −10 log (P), where P is the probability that the observed match is a random event. The identification is considered to be valid if the MOWSE score is greater than or equal to the significance threshold (P < 0.05). This list includes only those proteins identified with a, pI, isoelectric point as calculated from the identified protein sequence, sequence coverage is the % of sequence of the matching peptides and category represents the grouping of western blotting signal (I-Individual antigen, II-genotype-specific antigen, III-common antigen, IV-Pathogenic specific for the species included in this study, V-species specific antigen, and VI-Unspecified antigen). (A) MS/MS product ion search result and (B) MS (PMF) ion search result. [file Table1.DOCX]

**Table S1:** List of proteins identified by MALDI TOF MS. Spot ID is the spot excised based on the western blot signals where L and P represents the signals out of serum L and P respectively and K represents the signals obtained from both of the sera with results from earlier study. Gel positions of spot numbers are shown in supplemental figures S1-S5. Gel represents 2D gel from where spots were excised, *P. zopfii* GT1 (SAG 2063), *P. zopfii* GT2 (SAG SAG 2021, PZ-L, PZ-P) and *P. blaschkeae* (SAG 2064). NCBI Acc. No. is the accession number of the proteins identified by comparing the peptide sequence with NCBI databank, Uniprot Acc. No. is the accession number of the proteins with Uniprot. MW: molecular weight calculated from identified protein sequence, The MOWSE score (MOlecular Weight SEarch score) is calculated by –10 log (P), where P is the probability that the observed match is a random event. The identification is considered to be valid if the MOWSE score is greater than or equal to the significance threshold (P<0.05). This list includes only those proteins identified with a, pI: isoelectric point as calculated from the identified protein sequence, sequence coverage is the % of sequence of the matching peptides and category represents the grouping of western blotting signal (I-Individual antigen, II-genotype-specific antigen, III-common antigen, IV-Pathogenic specific for the species included in this study, V-species specific antigen and VI-Unspecified antigen). A) MS/MS product ion search result and B) MS (PMF) ion search result.

| **Spot ID** | **Gel** | **NCBI Acc. No.** | **Uniprot Acc. No.** | **Protein** | **Organism** | **MW**  **(kDa)** | **MOWSE Score** | **peptides sequence** | **pI** | **Sequence cov. (%)** | **Category** | **Function** | |
| --- | --- | --- | --- | --- | --- | --- | --- | --- | --- | --- | --- | --- | --- |
| **A) MS/MS product ion** **search result** | | | | | | | | | | | | | |
| L2 | SAG 2021 | [gi\|493436781](http://www.matrixscience.com/cgi/protein_view.pl?file=..%2Fdata%2F20140916%2FFTgcfbawE.dat&hit=1&db_idx=1) |  | RNA polymerase sigma54 factor | *Achromobacter xylosoxidans* | 49153 | 90 | R.GWQVTLNSAAVPR.L R.RGWQVTLNSAAVPR.L | 4,90 | 17 | VI | transcription start of enzymes for arginin degradation, expressed by lack of nitrogen | |
| L5 | SAG 2021 | [gi\|81237625](http://www.matrixscience.com/cgi/protein_view.pl?file=..%2Fdata%2F20140627%2FFTnrlzsmR.dat&hit=3&db_idx=1) |  | 14-3-3 protein | *Dunaliella salina* | 29437 | 126 | K.AAQDIALVDLPPTHPIR.L | 4,95 | 6 | III |  | |
| L9=P9** | SAG 2021 | [gi\|515707702](http://www.matrixscience.com/cgi/protein_view.pl?file=..%2Fdata%2F20140106%2FFTnmoGeSh.dat&hit=1) |  | serine recombinase | *Rhodobacter sphaeroides* | 57111 | 87 | R.EIAKILNADGHR.T | 7,12 | 33 | II | genetic recombination | |
| L12 =P7 | SAG 2021 | [gi\|146098508](http://www.matrixscience.com/cgi/protein_view.pl?file=..%2Fdata%2F20140627%2FFTnrlzutS.dat&hit=1&db_idx=1) | [A4I9I3](http://www.uniprot.org/uniprot/A4I9I3) | malate dehydrogenase | *Leishmania infantum* | 33659 | 115 | R.LFGVTTLDVVR.A K.RLFGVTTLDVVR.A | 8,86 | 16 | II | enzyme of metabolic pathways e.g. citric acid cicle | |
| L16*** | SAG 2021 | gi\|342218495 | F9MPS9 | aspartate semialdehyde dehydrogenase | *Megasphaera sp* | 38191 | 92 | MKKPVVAILGATGAVGQEFIR.L K.PVVAILGATGAVGQEFIR.L K.EGIEELETEVK.D K.DLYNEKAVNLWVCGDQIR.K | 5,60 | 14 | VI | biosynthesis of amino acids | |
| L17*** | SAG 2021 | gi\|226227133 | C1A3U4 | anthranilate phosphoribosyltransferase | *Gemmatimonas aurantiaca* | 35459 | 61 | Top of Form  K.GETPSEVAAVVR.ABottom of Form | 6.00 | 3 | VI | biosynthesis of aromatic amino acids | |
| L19 | PZ-L | gi\|322694421 | [E9ECV7](http://www.uniprot.org/uniprot/E9ECV7) | hypothetical protein MAC_07705 | *Metarhizium acridum* | 36993 | 89 | R.DGMMLGAALSGLEINFIDGVR.G + 2 Oxidation (M) | 6,34 | 28 | I |  | |
| L24 | PZ-L | gi\|350272363 | [G4KYG2](http://www.uniprot.org/uniprot/G4KYG2) | flagellar motor switch protein FliG | *Oscillibacter valericigenes* | 40515 | 101 | K.MESPSPAAMKIIETEMGR.R + Oxidation (M) | 5,09 | 34 | VI | chemotaxis | |
| L29 = P53 | PZ-L | [gi\|552840509](http://www.matrixscience.com/cgi/protein_view.pl?file=..%2Fdata%2F20140804%2FFTgArnant.dat&hit=1&db_idx=1) | [E1Z7R4](http://www.uniprot.org/uniprot/E1Z7R4) | heat shock protein 70 | *Chlorella variabilis* | 71207 | 164 | R.FEELNMDLFR.K R.TTPSYVAFTDTER.L R.ARFEELNMDLFR.K K.AVITVPAYFNDSQR.Q | 5,41 | 17 | II | protein family, chaperon system, extracellular: proinflammatory effects | |
| L30 | PZ-L | [gi\|598036021](http://www.matrixscience.com/cgi/protein_view.pl?file=..%2Fdata%2F20140917%2FFTgcraHmh.dat&hit=2&db_idx=1) | [J0WXZ5](http://www.uniprot.org/uniprot/J0WXZ5) | hypothetical protein AURDEDRAFT_125187 | *Auricularia delicata* | 56444 | 87 | K.LRKSGQSAAPTHVR.K | 9,09 | 25 | II |  | |
| L33 | PZ-L | [gi\|491671806](http://www.matrixscience.com/cgi/protein_view.pl?file=..%2Fdata%2F20140918%2FFTgcrbctS.dat&hit=1&db_idx=1) |  | pyridoxal biosynthesis lyase | *Corynebacterium striatum* | 29352 | 87 | -.MLKGGVIMDVVTPEQAK.I + Oxidation (M) | 4,88 | 39 | VI | Cofactor biosynthesis; pyridoxal 5'-phosphate biosynthesis.lyase activity | |
| L36 | PZ-L | [gi\|651251926](http://www.matrixscience.com/cgi/protein_view.pl?file=..%2Fdata%2F20140918%2FFTgcrbaSR.dat&hit=1&db_idx=1) |  | glyceraldehyde-3-phosphate dehydrogenase | *Acidobacteria bacterium* | 35909 | 94 | K.IISWYDNEWGYSNR.V | 7,74 | 11 | III | glycolysis | |
| L37**** | PZ-L | gi\|15802193 | [A0A075U8E2 P0A9B4](http://www.uniprot.org/uniprot/A0A075U8E2) | glyceraldehyde-3-phosphate dehydrogenase | *Escherichia coli* | 35510 | 163 | K.LTGMAFR.V R.GASQNIIPSSTGAAK.A R.GASQNIIPSSTGAAK.A K.FGIETGLMTTVHATTATQK.T K.FGIETGLMTTVHATTATQK.T | missing, identified by irontrap | | III | glycolysis | |
| L39 | PZ-L | [gi\|495015358](file:///C:\Users\jayaseelan\AppData\Local\Microsoft\Windows\Temporary%20Internet%20Files\Content.MSO\90EBAF1E.tmp#RANGE!Hit1) | [W6WZG1](http://www.uniprot.org/uniprot/W6WZG1) | PAS domain S-box | *Burkholderia* sp. | 35397 | 86/92* | K.MPGIDGFETCRR.L | 5,96 | 33 | II | cell signalling | |
| L44 | PZ-P | [gi\|489383894](http://www.matrixscience.com/cgi/protein_view.pl?file=..%2Fdata%2F20140918%2FFTgcrbawt.dat&hit=1&db_idx=1) |  | ATP-dependent helicase | *Pseudomonas stutzeri* | 91410 | 90 | R.LANGRAAQFDEPDALMK.E R.VGELVLAREPLPQLDDDAR.G | 6,26 | 20 | VI | transkription/replication | |
| L45 | PZ-P | [gi\|494277650](http://www.matrixscience.com/cgi/protein_view.pl?file=..%2Fdata%2F20140804%2FFTgArnTEe.dat&hit=1&db_idx=1) |  | LysR family transcriptional regulator | *Pseudomonas psychrotolerans* | 33913 | 99 | R.GVLQVAAPSDLGR.N | 9.00 | 43 | III | procaryotic transcriptional regulator i.a. virulence | |
| L46 = P70 | PZ-P | [gi\|501190284](http://www.matrixscience.com/cgi/protein_view.pl?file=..%2Fdata%2F20140917%2FFTgcraHwm.dat&hit=1&db_idx=1) | [A9EN65](http://www.uniprot.org/uniprot/A9EN65) | hypothetical protein | *Sorangium cellulosum* | 62923 | 91 | R.WAVRGLGAMKSEK.A + Oxidation (M) | 6,95 | 24 | V |  | |
|  | PZ-P | [gi\|652870124](http://www.matrixscience.com/cgi/protein_view.pl?file=..%2Fdata%2F20140917%2FFTgcraHwm.dat&hit=2&db_idx=1) |  | transcription elongation factor NusA | *Gaetbulibacter saemankumensis* | 46561 | 89 | R.IDPVGACVGMKGSR.I + Oxidation (M) | 4,68 | 33 |  | elongation factor, DNA repaire function, stress response | |
| L51 | PZ-P | [gi\|576424619](http://www.matrixscience.com/cgi/protein_view.pl?file=..%2Fdata%2F20140807%2FFTgAreTSh.dat&hit=1&db_idx=1) | [X7ZFM4](http://www.uniprot.org/uniprot/X7ZFM4) | short chain dehydrogenase family protein | *Mycobacterium xenopi* | 16881 | 92* | R.TIGVVSRITPLGD.- | 9,78 | 51 | VI | big enzyme family, u.a. oxidoreductase, Coeenzym abhängig (NAD o. NADP) | |
| L52 peptide | PZ-P | [gi\|449444152](http://www.matrixscience.com/cgi/protein_view.pl?file=..%2Fdata%2F20140918%2FFTgcrxYaL.dat&hit=gi%7C449444152&db_idx=1&px=1&ave_thresh=61&_ignoreionsscorebelow=0&report=20&_sigthreshold=0.05&_msresflags=1025&_msresflags2=2&percolate=-1&percolate_rt=0&_minpeplen=7&sessionID=guest_guestsession) |  | glyceraldehyde-3-phosphate dehydrogenase | *Cucumis sativus* | 45131 | 63 | K.LISWYDNEWGYSNR.V | 8,50 | 3 | III | glycolysis | |
| L54 | PZ-P | [gi\|89112882](http://www.matrixscience.com/cgi/protein_view.pl?file=..%2Fdata%2F20140515%2FFTneSrcTm.dat&hit=2) | [A1XIU4](http://www.uniprot.org/uniprot/A1XIU4) | ATPase alpha subunit | *Passiflora suberosa* | 40128 | 132 | K.AVDSLVPIGR.G R.EAFPGDVFYLHSR.L | 8,50 | 20 | II | Energy metabolism | |
| L56 | PZ-P | [gi\|89329739](file:///C:\Users\jayaseelan\AppData\Local\Microsoft\Windows\Temporary%20Internet%20Files\Content.MSO\90EBAF1E.tmp#RANGE!Hit1) | [Q09TP7](http://www.uniprot.org/uniprot/Q09TP7) | heat shock protein 70, partial | *Capsaspora owczarzaki* | 53525 | 122 | K.GTIDDIVLVGGSTR.I R.TTPSYVAFTDTER.L K.STAGDTHLGGEDFDNR.M K.MDKGTIDDIVLVGGSTR.I | 5,57 | 12 | II | protein family, chaperon system, extracellular: proinflammatory effects | |
| L57 | SAG 2063 | [gi\|347548269](http://www.matrixscience.com/cgi/protein_view.pl?file=..%2Fdata%2F20140804%2FFTgArnHSh.dat&hit=1&db_idx=1) | [G2ZAH1](http://www.uniprot.org/uniprot/G2ZAH1) | putative phosphomannomutase | *Listeria ivanovii subsp. ivanovii* | 62483 | 91 | R.ELTVCYTPLHGAGK.E | 5,62 | 27 | VI | glycosylation of proteins | |
| L72 | SAG 2063 | [gi\|567866037](http://www.matrixscience.com/cgi/protein_view.pl?file=..%2Fdata%2F20140805%2FFTgArraaS.dat&hit=1&db_idx=1) | [V4SP65](http://www.uniprot.org/uniprot/V4SP65) | hypothetical protein CICLE_v10024708mg | *Citrus clementina* | 163841 | 103 | K.TLKEACEQGDAEKR.L | 5,33 | 13 | V |  | |
| L73 peptide | SAG 2063 | [gi\|73853033](http://www.matrixscience.com/cgi/protein_view.pl?file=..%2Fdata%2F20140807%2FFTgAreTee.dat&hit=gi%7C73853033&db_idx=1&px=1&ave_thresh=58&_ignoreionsscorebelow=0&report=20&_sigthreshold=0.05&_msresflags=1025&_msresflags2=2&percolate=-1&percolate_rt=0&_minpeplen=7&sessionID=guest_guestsession) | [Q3ZUA9](http://www.uniprot.org/uniprot/Q3ZUA9) | glyceraldehyde-3-phosphate dehydrogenase | *Staphylococcus aureus subsp. aureus* | 3587 | 64* | -.WYDNEWGYSNR.V | 4,18 | 34 | III | glycolysis | |
| L75 peptide | SAG 2063 | [gi\|73853033](http://www.matrixscience.com/cgi/protein_view.pl?file=..%2Fdata%2F20140805%2FFTgArruST.dat&hit=gi%7C73853033&db_idx=1&px=1&ave_thresh=58&_ignoreionsscorebelow=0&report=20&_sigthreshold=0.05&_msresflags=1025&_msresflags2=2&percolate=-1&percolate_rt=0&_minpeplen=7&sessionID=guest_guestsession) | [Q3ZUA9](http://www.uniprot.org/uniprot/Q3ZUA9) | glyceraldehyde-3-phosphate dehydrogenase | *Staphylococcus aureus subsp. aureus* | 3587 | 59 | -.WYDNEWGYSNR.V | 4,18 | 34 | III | glycolysis | |
| L82 | SAG 2064 | [gi\|405973044](http://www.matrixscience.com/cgi/protein_view.pl?file=..%2Fdata%2F20140919%2FFTgcoGcOt.dat&hit=1&db_idx=1) | [K1RT53](http://www.uniprot.org/uniprot/K1RT53) | hypothetical protein CGI_10023543 | *Crassostrea gigas* | 14739 | 94 | K.KALSVVIAVGSEHFQSIRK.G | 9,07 | 51 | VI |  | |
| L90 | SAG 2064 | [gi\|566226950](http://www.matrixscience.com/cgi/protein_view.pl?file=..%2Fdata%2F20140805%2FFTgArrene.dat&hit=1&db_idx=1) | [W1UBZ5](http://www.uniprot.org/uniprot/W1UBZ5) | hypothetical protein Q607_CBUC00038G0011 | *Clostridium butyricum* | 10285 | 88 | K.ICKDTSNASMK.R | 9,67 | 55 | VI |  | |
| L97 | SAG 2064 | [gi\|353604882](http://www.matrixscience.com/cgi/protein_view.pl?file=..%2Fdata%2F20140805%2FFTgArrTTR.dat&hit=1&db_idx=1) | [G5NYX9](http://www.uniprot.org/uniprot/G5NYX9) | Regulatory protein CII | *Salmonella enterica subsp. enterica serovar Johannesburg str.* | 18907 | 87 | R.MLSLSALALHAR.L K.HTMIESVNSGIR.M + Oxidation (M) | 6,36 | 27 | III | regulation of transcription, proteolysis of Lambda-phage | |
| L98a | SAG 2064 | [gi\|563553400](http://www.matrixscience.com/cgi/protein_view.pl?file=..%2Fdata%2F20140808%2FFTgAoGcee.dat&hit=1&db_idx=1) | [X6D4Z8](http://www.uniprot.org/uniprot/X6D4Z8) | hydrolase | *Mesorhizobium* sp. | 32706 | 92* | R.RAVDRAAEFMTEAR.H + Oxidation (M) R.QLAQAPVPTLVIHAPDDR.E | 10,84 | 33 | III | catabolism | |
| L103 | SAG 2064 | [gi\|398025495](http://www.matrixscience.com/cgi/protein_view.pl?file=..%2Fdata%2F20140805%2FFTgArrHES.dat&hit=1&db_idx=1) | [R4IFH9](http://www.uniprot.org/uniprot/R4IFH9) | nucleoprotein, partial | *Lleida bat lyssavirus* | 31545 | 94 | R.MEQIFETAPFAK.I + Oxidation (M) | 5,91 | 25 | III |  | |
| L104 | SAG 2064 | [gi\|374316192](http://www.matrixscience.com/cgi/protein_view.pl?file=..%2Fdata%2F20140805%2FFTgArrHeS.dat&hit=1&db_idx=1) | [G8QY45](http://www.uniprot.org/uniprot/G8QY45) | response regulator with putative antiterminator output domain | *Sphaerochaeta pleomorpha str. Grapes* | 21514 | 86/89* | K.QLFFQTLGLARSMRR.R + Oxidation (M) | 6,62 | 27 | III | RNA binding | |
| K | SAG 2021 | [gi\|224593225](http://www.matrixscience.com/cgi/protein_view.pl?file=..%2Fdata%2F20140307%2FFTnuonHEE.dat&hit=1) | [C0LL61](http://www.uniprot.org/uniprot/C0LL61) | elongation factor 1 alpha | *Parachlorella kessleri* | 34932 | 159 | R.LLFELGGIPER.E R.WHYTIIDAPGHR.D | 7,88 | 32 | III | translation | |
| P14*** | SAG 2021 | gi\|34222896 | Q8KCH7 | Triosephosphate isomerase | *Chlorobium tepidum* | 26282 | 53 | Top of Form  K.LVIAYEPVWAIGTGK.TBottom of Form | 4,85 | 6 | III | glycolysis | |
| P22 | SAG 2021 | [gi\|499247019](http://www.matrixscience.com/cgi/protein_view.pl?file=..%2Fdata%2F20140922%2FFTgcoicST.dat&hit=1&db_idx=1) | [Q7VNG5](http://www.uniprot.org/uniprot/Q7VNG5) | ATP-dependent DNA helicase Rep | *Haemophilus ducreyi* | 78034 | 94 | R.GNHQSRLLEKTLMQNR.I + Oxidation (M) | 5,98 | 20 | VI | transkription/replication | |
| P25 | SAG 2021 | [gi\|498459689](http://www.matrixscience.com/cgi/protein_view.pl?file=..%2Fdata%2F20140805%2FFTgArrYnS.dat&hit=1&db_idx=1) |  | N6-adenine-specific DNA methylase | *Enterococcus moraviensis* | 43905 | 97 | K.GGAPLKENMAAALVALTNWRK.D K.EYGVMVANPPYGERLGEEESVRK.L | 5,50 | 31 | VI | replication | |
| P27 | SAG 2021 | [gi\|658007268](http://www.matrixscience.com/cgi/protein_view.pl?file=..%2Fdata%2F20140918%2FFTgcrzunh.dat&hit=1&db_idx=1) |  | PREDICTED: uncharacterized protein LOC103401876 | *Malus domestica* | 26519 | 89 | R.QVIVNSNNGGSGPR.I R.RQVIVNSNNGGSGPR.I | 10,38 | 32 | VI |  | |
| P29*** | SAG 2021 | gi\|3059122 | O65843 | glyceraldehyde-3-phosphate dehydrogenase | *Marsilea quadrifolia* | 39352 | 57 | K.LVSWYDNEWGYSNR.V | 8,69 | 3 | V | glycolysis | |
| P30 | SAG 2021 | [gi\|518001895](http://www.matrixscience.com/cgi/protein_view.pl?file=..%2Fdata%2F20140805%2FFTgArrSeS.dat&hit=1&db_idx=1) |  | DeoR family transcriptional regulator | *Pseudaminobacter salicylatoxidans* | 34086 | 88 | R.AGWLYYVAGNTQDQIAGKLGVSR.Q | 5,78 | 29 | VI | transcription | |
| P33 | PZ-L | [gi\|477524468](http://www.matrixscience.com/cgi/protein_view.pl?file=..%2Fdata%2F20140805%2FFTgArrSem.dat&hit=1&db_idx=1) | [N4UMH9](http://www.uniprot.org/uniprot/N4UMH9) | malate dehydrogenase | *Colletotrichum orbiculare* | 35328 | 94 | R.LFGVTTLDVVR.A | 8,64 | 17 | II | enzyme of metabolic pathways e.g. citric acid cicle | |
| P36 | PZ-L | [gi\|566028571](http://www.matrixscience.com/cgi/protein_view.pl?file=..%2Fdata%2F20140808%2FFTgAoGumT.dat&hit=1&db_idx=1) | [V9FM53](http://www.uniprot.org/uniprot/V9FM53) | hypothetical protein F443_05159 | *Phytophthora parasitica* | 43755 | 93* | R.MQELAMLIGALTK.S + Oxidation (M) K.QKLSGIQNTTSSLGRSLMEMVLLLR.E + 2 Oxidation (M) | 5,71 | 27 | II |  | |
| P37 | PZ-L |  |  | hypothetical protein | *Clostridium* sp. | 23482 | 87 | R.EFGSGGRELGKR.L | 6,11 | 24 | VI |  | |
| P42 | PZ-L | [gi\|545454000](file:///C:\Users\jayaseelan\AppData\Local\Microsoft\Windows\Temporary%20Internet%20Files\Content.MSO\90EBAF1E.tmp#RANGE!Hit1) |  | putative oxidoreductase | *Novosphingobium tardaugens* | 27193 | 86 | R.QIAADVGKYNIR.V | 4,86 | 42 | III | metabolism | |
| P42 peptide | PZ-L | [gi\|255089781](http://www.matrixscience.com/cgi/protein_view.pl?file=..%2Fdata%2F20140808%2FFTgAoGuaL.dat&hit=gi%7C255089781&db_idx=1&px=1&ave_thresh=60&_ignoreionsscorebelow=0&report=20&_sigthreshold=0.05&_msresflags=1025&_msresflags2=2&percolate=-1&percolate_rt=0&_minpeplen=7&sessionID=guest_guestsession) | [C1EIT5](http://www.uniprot.org/uniprot/C1EIT5) | predicted protein | *Micromonas* sp. | 38248 | 68* | R.TRGVVHAHADAVR.S | 5,75 | 3 |  |  | |
| P45 | PZ-L | [gi\|490188060](http://www.matrixscience.com/cgi/protein_view.pl?file=..%2Fdata%2F20140806%2FFTgArfHmS.dat&hit=1&db_idx=1) |  | similar to Antirestriction protein, partial | *Xylella fastidiosa* | 157358 | 87 | K.QQAIAQNPEPMR.R + Oxidation (M) K.QQAIAQNPEPMRR.Q + Oxidation (M) | 5,57 | 14 | VI | degradation of extrinsic DANN | |
| P53 | PZ-L | gi\|330791727 | [F0Z957](http://www.uniprot.org/uniprot/F0Z957) | hypothetical protein DICPUDRAFT_4515 | *Dictyostelium purpureum* | 71719 | 102 | R.ATNGDTFLGGEDFDNELLK.Y | 5,75 | 20 | V |  | |
| P54 | PZ-L | [gi\|668347690](http://www.matrixscience.com/cgi/protein_view.pl?file=..%2Fdata%2F20141104%2FFTgolrESE.dat&hit=6&db_idx=1) | [A0A077JFW5](http://www.uniprot.org/uniprot/A0A077JFW5) | transposase, partial | *Escherichia coli* | 35990 | 89 | K.WTTDYNRRSIAETAMYR.V + Oxidation (M) | 10,08 | 36 | V | DNA mobilisation | |
| P56 | PZ-P | gi\|495330387 | [H1WI36](http://www.uniprot.org/uniprot/H1WI36) | hypothetical protein | *Arthrospira* sp. | 10506 | 89 | M.LSMTGQLLNVFENPR.V + Oxidation (M) | 7,90 | 42 | II |  | |
| P57 peptide | PZ-P | [gi\|653033880](http://www.matrixscience.com/cgi/protein_view.pl?file=..%2Fdata%2F20140918%2FFTgcrzeSL.dat&hit=gi%7C653033880&db_idx=1&px=1&ave_thresh=61&_ignoreionsscorebelow=0&report=20&_sigthreshold=0.05&_msresflags=1025&_msresflags2=2&percolate=-1&percolate_rt=0&_minpeplen=7&sessionID=guest_guestsession) |  | phosphonate ABC transporter permease | *Rubritepida flocculans* | 26192 | 63 | R.IAEVQPLALVDGLPR.L | 8,97 | 6 | II | organic phosphonate transport | |
| P59 | PZ-P | [gi\|665970080](http://www.matrixscience.com/cgi/protein_view.pl?file=..%2Fdata%2F20140922%2FFTgcoiume.dat&hit=1&db_idx=1) |  | hypothetical protei | *Rhodopseudomonas* sp. | 40508 | 88 | R.LTGMLSQFNRSGIVQDMVHR.L + Oxidation (M) | 6,62 | 45 | VI |  | |
| P60 | PZ-P | gi\|498154438 |  | hypothetical protei | *Acaryochloris* sp. | 26777 | 93 | R.GSGQPLNAGLQQSMGQAMR.A + 2 Oxidation (M) | 8,34 | 20 | VI |  | |
| P64 | PZ-P | [gi\|490077145](http://www.matrixscience.com/cgi/protein_view.pl?file=..%2Fdata%2F20140918%2FFTgcrzene.dat&hit=1&db_idx=1) |  | 3-dehydroquinate synthase | *Streptomyces rimosus* | 39438 | 89 | K.VSPGRPSFVLSPR.R K.VSPGRPSFVLSPRR.G | 9,68 | 31 | I | synthesis of aromatic amino acids | |
| P65 | PZ-P | [gi\|573891266](http://www.matrixscience.com/cgi/protein_view.pl?file=..%2Fdata%2F20140806%2FFTgAriSat.dat&hit=1&db_idx=1) |  | PREDICTED: ankyrin repeat domain-containing protein 26-like | *Lepisosteus oculatus* | 308505 | 107* | R.ILNIPVKVNKAEVNFDK.N | 4,90 | 6 | VI | unknown | |
| P66 | PZ-P | [gi\|495682127](http://www.matrixscience.com/cgi/protein_view.pl?file=..%2Fdata%2F20140806%2FFTgAriStE.dat&hit=1&db_idx=1) | [K1V9F5](http://www.uniprot.org/uniprot/K1V9F5) | HrpA-like helicase, partial | *Streptomyces* sp. | 62478 | 90 | R.RLLPWAGGEAGR.L R.AGDGYLMVSGTGAELGPGSALRESR.W + Oxidation (M) | 10,16 | 16 | III | transkription/replication | |
| P69 | PZ-P | [gi\|586633520](http://www.matrixscience.com/cgi/protein_view.pl?file=..%2Fdata%2F20140806%2FFTgArxcnO.dat&hit=1&db_idx=1) | [W7WV59](http://www.uniprot.org/uniprot/W7WV59) | protocatechuate 4,5-dioxygenase subunit alpha | *Hydrogenophaga* sp. | 10347 | 90* | M.SKTIPGTTPFDGDMAKK.A + Oxidation (M) | 9,81 | 54 | III | PCA pathway (breakdown of lignin) | |
| P74 | SAG 2063 | [gi\|594028872](http://www.matrixscience.com/cgi/protein_view.pl?file=..%2Fdata%2F20140806%2FFTgArxaOm.dat&hit=3&db_idx=1) | [W8R1U1](http://www.uniprot.org/uniprot/W8R1U1) | membrane protein | *Pseudomonas stutzeri* | 43220 | 91 | K.LQDSLNGMFGGRK.R + Oxidation (M) | 5,28 | 22 | VI | cell structure | |
| P89 | SAG 2063 | [gi\|550991193](http://www.matrixscience.com/cgi/protein_view.pl?file=..%2Fdata%2F20140807%2FFTgArzSam.dat&hit=1&db_idx=1) | [U5N8Q3](http://www.uniprot.org/uniprot/U5N8Q3) | glutamate synthase small subunit | *Candidatus Symbiobacter mobilis* | 54179 | 89* | R.AAEVLHGTNNFPEITGR.I | 6,45 | 16 | VI |  | |
| P90 | SAG 2063 | [gi\|517468219](http://www.matrixscience.com/cgi/protein_view.pl?file=..%2Fdata%2F20140807%2FFTgArzStR.dat&hit=1&db_idx=1) |  | hypothetical protein | *Frankia* sp. | 155627 | 90 | R.MARAAPAGLGPVR.D + Oxidation (M) | 5,33 | 14 | VI |  | |
| P102 | SAG 2063 | [gi\|390343778](http://www.matrixscience.com/cgi/protein_view.pl?file=..%2Fdata%2F20140807%2FFTgArbaat.dat&hit=1&db_idx=1) | [W4XK99](http://www.uniprot.org/uniprot/W4XK99) | PREDICTED: protein BMH2 isoform 1 | *Strongylocentrotus purpuratus* | 29792 | 94* | R.KSASDQSLAAYK.T | 4,80 | 30 | III | 14-3-3 protein | |
| P103 | SAG 2063 | [gi\|558109608](http://www.matrixscience.com/cgi/protein_view.pl?file=..%2Fdata%2F20140807%2FFTgArbath.dat&hit=1&db_idx=1) | [K7GIN6](http://www.uniprot.org/uniprot/K7GIN6) | PREDICTED: glutamyl-tRNA(Gln) amidotransferase subunit B, mitochondrial | *Pelodiscus sinensis* | 60990 | 102 | R.RAQAAGPIASGQR.A | 8,99 | 18 | VI | glutamate biosynthesis | |
| P104 | SAG 2063 | [gi\|498981012](http://www.matrixscience.com/cgi/protein_view.pl?file=..%2Fdata%2F20140807%2FFTgArbume.dat&hit=1&db_idx=1) |  | PREDICTED: serine-rich adhesin for platelets-like isoform X4 | *Maylandia zebra* | 244277 | 88 | R.RRPSEDGKLPK.I | 5,32 | 5 | VI | adhesian, virulence factor of endovascular infections | |
| P104**** | SAG 2063 | gi\|552840509 |  | heat shock protein 70 | *Chlorella variabilis* | 70979 | 165 | R.LSKDEIER.M R.ARFEELNMDLFR.K K.AVITVPAYFNDSQR.Q | 5,41 | 17 |  | protein family, chaperon system, extracellular: proinflammatory effects | |
| P105 | SAG 2063 | [gi\|336117525](http://www.matrixscience.com/cgi/protein_view.pl?file=..%2Fdata%2F20140807%2FFTgArbswO.dat&hit=2&db_idx=1) | [F5XT18](http://www.uniprot.org/uniprot/F5XT18) | hypothetical protein | *Microlunatus phosphovorus* | 6686 | 88* | .MIPSTSASNKAGR.I + Oxidation (M) R.AHDPGSTKALSANR.S | 9,69 | 93 | VI |  | |
| P109 | SAG 2064 | [gi\|515112118](http://www.matrixscience.com/cgi/protein_view.pl?file=..%2Fdata%2F20140807%2FFTgArbemT.dat&hit=1&db_idx=1) |  | alveolysin | *Brevibacillus brevis* | 56507 | 87 | R.TIYVKLETTSK.S K.KSLSTSPVDISIIDSMANR.T | 5,05 | 17 | VI | Toxin | |
| P110 | SAG 2064 | [gi\|571046342](http://www.matrixscience.com/cgi/protein_view.pl?file=..%2Fdata%2F20140807%2FFTgArbeaE.dat&hit=1&db_idx=1) | [V9Z6P3](http://www.uniprot.org/uniprot/V9Z6P3) | hypothetical protein | *Streptomyces* sp. | 45931 | 87 | R.EVQPGAHSHRHLAKMVK.K + Oxidation (M) | 6,28 | 24 | VI |  | |
| P111 | SAG 2064 | [gi\|655153503](http://www.matrixscience.com/cgi/protein_view.pl?file=..%2Fdata%2F20140922%2FFTgcoxsmR.dat&hit=1&db_idx=1) |  | plasmid stabilization protein | *Paenibacillus pasadenensis* | 11363 | 112 | R.KTGDLSGLFGYDVSYK.E | 6,59 | 78 | VI | regulation of transcription | |
|  | SAG 2064 | [gi\|548069985](http://www.matrixscience.com/cgi/protein_view.pl?file=..%2Fdata%2F20140807%2FFTgArbTEL.dat&hit=1&db_idx=1) | [R7C6B5](http://www.uniprot.org/uniprot/R7C6B5) | putative bacterial extracellular solute-binding protein | *Clostridium* sp. | 117445 | 97 | K.GLWNFGMVPGVK.Q K.NMVDQQFTPTSK.E + Oxidation (M) K.KIPTYAEYAAENK.G | 5,01 | 10 | VI | transmembrane transport | |
| P115 | SAG 2064 | [gi\|556498531](http://www.matrixscience.com/cgi/protein_view.pl?file=..%2Fdata%2F20140807%2FFTgArbHEm.dat&hit=1&db_idx=1) | [V2YG55](http://www.uniprot.org/uniprot/V2YG55) | hypothetical protein | *Firmicutes bacterium* | 180016 | 94* | R.TRPNGTFYPNSR.M R.IKVPVFELENEKILNR.H | 6,40 | 5 | VI |  | |
| P122 | SAG 2064 | [gi\|432105093](http://www.matrixscience.com/cgi/protein_view.pl?file=..%2Fdata%2F20140807%2FFTgArbHth.dat&hit=1&db_idx=1) | [L5LZI5](http://www.uniprot.org/uniprot/L5LZI5) | Protein phosphatase Slingshot like protein 1 | *Myotis davidii* | 111373 | 88* | K.GAALFLQQGSSPQGQR.S K.ESALPAPVSALSSGSPERK.E R.NELEKQMNCNLKEFK.E + Oxidation (M) R.MHVFKPVSVQAMWSALQVLHK.A + 2 Oxidation (M) | 6,26 | 16 | VI | actin cytoskeleton organization | |
| P129 | SAG 2064 | [gi\|490045609](http://www.matrixscience.com/cgi/protein_view.pl?file=..%2Fdata%2F20140807%2FFTgAreamR.dat&hit=1&db_idx=1) |  | phosphate starvation protein PhoH | *Streptomyces albus* | 37720 | 87 | R.GNEISATGDPAEVALVQR.L | 6,35 | 28 | III | cell signalling | |
| P132 peptide | SAG 2064 | [gi\|21427320](http://www.matrixscience.com/cgi/protein_view.pl?file=..%2Fdata%2F20140918%2FFTgcrzHeO.dat&hit=gi%7C21427320&db_idx=1&px=1&ave_thresh=60&_ignoreionsscorebelow=0&report=20&_sigthreshold=0.05&_msresflags=1025&_msresflags2=2&percolate=-1&percolate_rt=0&_minpeplen=7&sessionID=guest_guestsession) | [Q8JHP5](http://www.uniprot.org/uniprot/Q8JHP5) | Hsp70 protein | *Mitsukurina owstoni* | 50707 | 70 | K.AVITVPAYFNDSQR.Q K.STAGDTHLGGEDFDNR.M | 8,58 | 6 | VI | protein family, chaperon system, extracellular: proinflammatory effects | |
| P135 | SAG 2064 | [gi\|498028995](http://www.matrixscience.com/cgi/protein_view.pl?file=..%2Fdata%2F20140807%2FFTgAreaOe.dat&hit=1&db_idx=1) |  | GntR family transcriptional regulator | *Xanthomonas* | 27975 | 93 | R.SADGRVMAYEK.A + Oxidation (M) R.GTPVVRALQYFR.A K.GGQWKPGEALPAER.Q | 8,51 | 28 | VI | transcription | |
| P136 | SAG 2064 | gi\|470114885 |  | PREDICTED: KH domain-containing protein At4g18375-like | *Fragaria vesca subsp. vesca* | 6955 | 91 | K.VIGKGGANIANIR.K | 10,16 | 94 | VI | unknown | |
|  | | | | | | | | | | | | |  |
| **B) MS (PMF) search result** | | | | | | | | | | | | | |
| L22 | PZ-L | [gi\|575768043](http://www.matrixscience.com/cgi/protein_view.pl?file=..%2Fdata%2F20140804%2FFTgArnaSh.dat&hit=1&db_idx=1) | [W4N3V9](http://www.uniprot.org/uniprot/W4N3V9) | beta-lactamase | *Acinetobacter baumannii* | 175839 | 88 |  | 9,07 | 35 | VI | resistance to antibiotics | |
| L32 | PZ-L | [gi\|553311399](http://www.matrixscience.com/cgi/protein_view.pl?file=..%2Fdata%2F20140917%2FFTgcraEnT.dat&hit=1&db_idx=1) | [U7UBR0](http://www.uniprot.org/uniprot/U7UBR0) | radical SAM protein, TIGR01212 | *Peptoniphilus* sp. | 39354 | 93 |  | 9,02 | 33 | V | metabolism, enzyme superfamily | |
| L62 | SAG 2063 | [gi\|501257597](http://www.matrixscience.com/cgi/protein_view.pl?file=..%2Fdata%2F20140919%2FFTgconYnt.dat&hit=1&db_idx=1) |  | oxidoreductase | *Acinetobacter baumannii* | 69606 | 105 |  | 6,35 | 15 | VI | metabolism | |
| L86 | SAG 2064 | [gi\|257804208](http://www.matrixscience.com/cgi/protein_view.pl?file=..%2Fdata%2F20140919%2FFTgcoGaOE.dat&hit=1&db_idx=1) | [C9A0B3](http://www.uniprot.org/uniprot/C9A0B3) | conserved hypothetical protein | *Enterococcus gallinarum* | 14442 | 88 |  | 8,61 | 68 | II |  | |
| P9 = L9 | SAG 2021 | [gi\|551556848](http://www.matrixscience.com/cgi/protein_view.pl?file=..%2Fdata%2F20140918%2FFTgcrzcwO.dat&hit=1&db_idx=1) | [R1BTN6](http://www.uniprot.org/uniprot/R1BTN6) | hypothetical protein EMIHUDRAFT_437153 | *Emiliania huxleyi* | 21320 | 88 |  | 10,79 | 42 | II | Translation, ubiquitär | |
| P10 | SAG 2021 | [gi\|488600113](http://www.matrixscience.com/cgi/protein_view.pl?file=..%2Fdata%2F20140805%2FFTgArrEnm.dat&hit=1&db_idx=1) | [N0BJE8](http://www.uniprot.org/uniprot/N0BJE8) | phenylalanyl-tRNA synthetase beta subunit | *Archaeoglobus sulfaticallidus* | 62771 | 101 |  | 4,97 | 39 | II | phenylalanyl-tRNA aminoacylatio | |
| P19 | SAG 2021 | [gi\|157117949](http://www.matrixscience.com/cgi/protein_view.pl?file=..%2Fdata%2F20140805%2FFTgArrYOO.dat&hit=1&db_idx=1) | [Q0IER9](http://www.uniprot.org/uniprot/Q0IER9) | procollagen-lysine,2-oxoglutarate 5-dioxygenase | *Aedes aegypti* | 82324 | 93 |  | 5,67 | 29 | VI | collagen biosynthesis | |
| P21 | SAG 2021 | [gi\|518353074](http://www.matrixscience.com/cgi/protein_view.pl?file=..%2Fdata%2F20140805%2FFTgArrYwS.dat&hit=1&db_idx=1) |  | elongation factor Ts | *Streptomyces* sp. | 29907 | 88 |  | 5,38 | 51 | VI | tranlsation, procaryotic | |
| P26 | SAG 2021 | [gi\|490895420](http://www.matrixscience.com/cgi/protein_view.pl?file=..%2Fdata%2F20140805%2FFTgArrYtE.dat&hit=1&db_idx=1) |  | hypothetical protein | *Acinetobacter* | 39370 | 107 |  | 7,68 | 31 |  |  | |
|  |  | [gi\|490820742](http://www.matrixscience.com/cgi/protein_view.pl?file=..%2Fdata%2F20140805%2FFTgArrYtE.dat&hit=2&db_idx=1) |  | competence protein ComL | *Acinetobacter parvus* | 38882 | 93 |  | 8,61 | 31 | II | DNA uptake | |
| P34 | PZ-L | [gi\|557825306](http://www.matrixscience.com/cgi/protein_view.pl?file=..%2Fdata%2F20140805%2FFTgArrSme.dat&hit=1&db_idx=1) |  | hypothetical protein | *Asticcacaulis* sp. | 5484 | 87 |  |  |  | VI |  | |
| P40 | PZ-L | [gi\|573534114](http://www.matrixscience.com/cgi/protein_view.pl?file=..%2Fdata%2F20140805%2FFTgArrSwR.dat&hit=1&db_idx=1) | [W4ANS5](http://www.uniprot.org/uniprot/W4ANS5) | siderophore synthetase component protein, siderophore-iron transmembrane transporter | *Paenibacillus* sp. | 68456 | 88 |  | 5,45 | 32 | VI | siderophore biosynthetic process | |
| P50 | PZ-L | gi\|350272363 |  | flagellar motor switch protein FliG | *Oscillibacter valericigenes* | 40515 | 89 |  | 5,09 | 31 | VI |  | |
| P51 | PZ-L | [gi\|322694421](http://www.matrixscience.com/cgi/protein_view.pl?file=..%2Fdata%2F20140806%2FFTgArfSeh.dat&hit=1&db_idx=1) |  | hypothetical protein MAC_07705 | *Metarhizium acridum* | 36993 | 95 |  | 6,34 | 38 | VI |  | |
|  | PZ-L | [gi\|220903845](http://www.matrixscience.com/cgi/protein_view.pl?file=..%2Fdata%2F20140806%2FFTgArfSeh.dat&hit=2&db_idx=1) | [B8IY83](http://www.uniprot.org/uniprot/B8IY83) | phosphate ABC transporter ATPase | *Desulfovibrio desulfuricans subsp. desulfuricans str.* | 28329 | 92 |  | 5,69 | 73 |  | Energy metabolism | |
| P80 | SAG 2063 | [gi\|406695927](http://www.matrixscience.com/cgi/protein_view.pl?file=..%2Fdata%2F20140807%2FFTgArzYOm.dat&hit=1&db_idx=1) | [K1V5I5](http://www.uniprot.org/uniprot/K1V5I5) | ribosomal protein | *Trichosporon asahii var. asahii* | 17044 | 91 |  | 10,49 | 67 | VI | translation | |
| P93 | SAG 2063 | [gi\|493507999](http://www.matrixscience.com/cgi/protein_view.pl?file=..%2Fdata%2F20140807%2FFTgArbaSh.dat&hit=1&db_idx=1) |  | nitrate/sulfonate/bicarbonate ABC transporter periplasmic protein | *Herbaspirillum frisingense* | 33967 | 88 |  | 9,32 | 26 | VI | transport | |
| P98 | SAG 2063 | [gi\|516861904](http://www.matrixscience.com/cgi/protein_view.pl?file=..%2Fdata%2F20140807%2FFTgArbaTe.dat&hit=1&db_idx=1) |  | hypothetical protein | *Acaricomes phytoseiuli* | 154279 | 94 |  | 6,31 | 17 | VI |  | |
| P130 | SAG 2064 | [gi\|241167622](http://www.matrixscience.com/cgi/protein_view.pl?file=..%2Fdata%2F20140807%2FFTgAreaOe.dat&hit=1&db_idx=1) | [B7PCQ4](http://www.uniprot.org/uniprot/B7PCQ4) | hypothetical protein IscW_ISCW003424 | *Ixodes scapularis* | 93969 | 94 |  | 7,91 | 15 | VI |  | |
| P134 | SAG 2064 | [gi\|497931242](http://www.matrixscience.com/cgi/protein_view.pl?file=..%2Fdata%2F20140923%2FFTgcoeTtT.dat&hit=1&db_idx=1) |  | hypothetical protein | *Peptoniphilus rhinitidis* | 65612 | 88* |  | 5,48 | 29 | III |  | |

| * | MS/MS tolerance of 1.2 |
| --- | --- |
| ** | Irrgang A, Weise C, Murugaiyan J, Roesler U. Identification of immunodominant proteins of the microalgae Prototheca by proteomic analysis. New Microbes New Infect. 2015;3:37–40. |
| *** | Murugaiyan J, Weise C, Bergen M von, Roesler U. Two-dimensional proteome reference map of *Prototheca zopfii* revealed reduced metabolism and enhanced signal transduction as adaptation to an infectious life style. Proteomics. 2013;13:2664–69. |
| **** | analysed by Iontrap |
